# Simulation-based training and education in laparoscopic hepatobiliary and pancreatic surgery

**DOI:** 10.3389/fsurg.2026.1787299

**Published:** 2026-04-01

**Authors:** Yaping Zhang, Jiliang Shen

**Affiliations:** 1 Department of Anesthesiology, Sir Run-Run Shaw Hospital, Zhejiang University School of Medicine, Hangzhou, Zhejiang, China; 2 Department of General Surgery, Sir Run-Run Shaw Hospital, School of Medical College, Zhejiang University, Hangzhou, China

**Keywords:** 3D-printed, augmented reality, surgical education, surgical simulation, virtual reality

## Abstract

Laparoscopic hepatobiliary and pancreatic (HBP) surgery necessitates mastery of intricate anatomical relationships and precision-dependent techniques. This review synthesizes advancements in simulation-based training and education modalities— three-dimensional (3D) printing, virtual reality (VR), augmented reality (AR). 3D-printed models provide unparalleled tactile fidelity for patient-specific anatomy replication but lack dynamic physiological responses. VR enables risk-free procedural repetition and AI-driven skill optimization, yet struggles with haptic authenticity. AR bridges preoperative planning and intraoperative execution through real-time holographic navigation but faces challenges in cognitive load management. Future advancements must prioritize hybrid simulation ecosystems integrating biomechanical realism with adaptive virtual interfaces, validated transfer-to-practice metrics, and ethical frameworks for emerging technologies.

## 1 Introduction

The paradigm shift toward minimally invasive techniques in hepatobiliary and pancreatic (HBP) surgery has exposed critical gaps in traditional surgical training and education. Laparoscopic pancreaticoduodenectomy, for instance, requires navigating a labyrinth of retroperitoneal vessels and ductal systems under restricted tactile feedback—a skill set poorly addressed by apprenticeship models reliant on opportunistic case exposure (1). Compounding this challenge are ethical concerns over live-patient training for high-risk procedures and the inconsistent availability of complex cases for skill maintenance. Simulation-based training has emerged as a transformative solution, offering three core advantages: (1) deliberate practice of critical scenarios without patient risk, (2) decomposition of complex procedures into modular competencies, and (3) objective performance assessment through sensor-based metrics and cognitive workload analysis (2). At present, the main methods of surgical simulation training include animal models, three-dimensional (3D) -printed physical surgical models and interactive models for virtual surgical training.

Porcine models continue to play an irreplaceable role in HBP surgical training due to their unparalleled physiological authenticity. Takahashi demonstrated that ex-vivo perfused porcine livers provide anatomically accurate training environments for laparoscopic procedures (3). The biliary systems of pigs closely resemble human counterparts in both structure and function, enabling realistic rehearsal of complex anastomotic techniques like hepaticojejunostomy. This anatomical compatibility allows trainees to develop tissue-handling skills that translate directly to clinical practice. McGuire emphasized the value of live porcine models in simulating intraoperative challenges (4). These systems allow surgeons to practice crisis management under conditions that mirror real-world stressors, such as hemorrhage control and unexpected vascular injuries. The physiological responses observed in these models closely align with human intraoperative dynamics, enhancing the validity of decision-making training. Animal surgical procedures necessitate coordinated collaboration among surgical, nursing, and anesthesiology teams, require established animal research facilities, and are further complicated by ethical regulations governing animal experimentation. These protocols involve logistically demanding preparations and are not amenable to frequent repetition within short timeframes.

Besides animal surgery simulation training, there have also emerged other simulation-based training methods. Among emerging technologies, 3D printing enables tactile exploration of patient-specific anatomy (5, 6), virtual reality (VR) provides immersive procedural rehearsal (7, 8), and augmented reality (AR) enhances intraoperative spatial reasoning (9, 10). This review critically evaluates these technologies through the lens of surgical education theory, proposes a unified training framework with integrated competency evaluation, and identifies key challenges for clinical implementation ().

## 2 3D-printed anatomical models

3D printing has significantly impacted surgical education, particularly in the realm of simulation-based training for laparoscopic hepatobiliary and pancreatic surgery. This technology allows for the creation of highly accurate anatomical models that can be used for preoperative planning, surgical simulation, and education. The integration of 3D printing into surgical training programs has been shown to enhance surgical skills and improve surgical outcomes (11).

One of the key benefits of 3D printing in surgical education is its ability to produce patient-specific models that replicate the unique anatomical features of individual patients (12). This capability is particularly useful in hepatobiliary and pancreatic surgery, where the anatomy can be highly variable and complex. For instance, the use of 3D-printed models in surgical planning for pancreaticobiliary diseases has been shown to improve the comprehension of pathoanatomical variations and aid in the adoption of appropriate surgical approaches (13). Furthermore, 3D printing has been utilized to create models for surgical simulation, allowing trainees to practice and refine their skills in a risk-free environment. This approach has been demonstrated to be effective in enhancing the proficiency of surgical trainees, as evidenced by studies showing that 3D-printed models can reduce perioperative complications in laparoscopic surgery for complex hepatobiliary diseases (14). Additionally, the use of 3D-printed models in surgical training has been associated with high levels of satisfaction among trainees, who appreciate the realism and educational value of these models (15). Our group developed cholangioenterostomy and pancreaticoenterostomy simulation models to facilitate rapid skill acquisition among inexperienced surgeons in performing these anastomotic procedures (16–18).

The currently available 3D-printed models employ commercially accessible printing materials and fail to take into account the texture and modulus of the organs (19, 20). Zhang developed a novel approach combining stereolithographic 3D printing with self-healing materials mimicking hepatic mechanical properties, enabling high-precision, repeatable surgical simulation while maintaining structural integrity at reduced cost (5). Addressing the limitations of 3D-printed models in simulating hemorrhage and other intraoperative scenarios, we developed a novel approach integrating 3D-printed vasculature with simulated blood perfusion, enabling realistic hemorrhage simulation during hepatic artery repair training (21).

Besides, Static models fail to simulate respiratory-induced liver movement or the pulsatile nature of the hepatic artery, creating a “time-frozen” representation of anatomy (22). Emerging trends focus on bioprinting technologies incorporating live hepatocytes and endothelial cells, potentially enabling physiological responses to surgical maneuvers—a frontier that could redefine preoperative planning within the next decade (23).

Compared with actual surgeries or high-end simulation equipment, 3D-printed models feature lower manufacturing costs and are thus suitable for large-scale application in training and teaching scenarios, though the actual cost can vary substantially depending on the complexity of anatomical segmentation, material grade, printing resolution, and post-processing refinements (24, 25). Previous studies have demonstrated that the average cost of a high-fidelity 3D-printed hepatic model is $152, which is far lower than the expense of animal experiments or high-end virtual reality (VR)/augmented reality (AR) simulation systems (24). In contrast to animal model-based training, simulation training using 3D-printed simulated organs is characterized by convenient model preparation and easy operation execution.

Nevertheless, the training method using 3D-printed models also has certain limitations. Pure 3D surgical model training focuses only on the operational practice of local surgical areas, but it is weak in restoring the overall intraoperative environment within the abdominal cavity. To a certain extent, it simplifies the difficulty and complexity of training and fails to deliver an immersive experience. It also has considerable limitations in the formation of visual memory for surgical procedures and the real-time evaluation of operational processes. Moreover, it lacks the function of real-time analysis and evaluation of suture angle, suture spacing and bimanual coordination during the operation.

Training with physical models often relies on expert evaluation of the operation process, which is restricted by the time and energy of experts. It is difficult to conduct one-on-one full-process quantitative evaluation and guidance in daily training. Instead, such training places more emphasis on the evaluation of suture results, while the assessment of trainees’ difficulties and challenges during training is insufficient, and targeted training guidance is even more lacking.

## 3 VR simulators

Simulation-based training, particularly through VR, provides a risk-free environment where surgical trainees can hone their skills without the immediate pressures and potential complications associated with real-life surgical procedures. The use of VR in surgical training allows for the replication of complex surgical scenarios, enabling trainees to practice and refine their techniques in a controlled setting.

One of the key advantages of VR simulation is its ability to provide a standardized training experience that can be tailored to the specific needs of the trainee. Studies have shown that VR simulation can effectively improve surgical skills, as it allows for repeated practice and immediate feedback, which are crucial for skill acquisition and retention (26, 27). High-fidelity systems like the LapSim-XR simulate the entire operative workflow of laparoscopic cholecystectomy, from port placement to cystic duct ligation, with real-time haptic feedback mimicking tissue resistance (28).

Moreover, VR simulation has been demonstrated to enhance the transfer of skills from the simulated environment to the operating room. For instance, a study comparing VR training with traditional methods found that trainees who underwent VR simulation performed better in real-life surgical tasks, indicating a positive transfer of skills (29). This transferability is critical in ensuring that the skills acquired through simulation are applicable and effective in actual surgical settings. AI systems incorporating reinforcement learning can enable closed-loop operation-feedback-optimization training, significantly enhancing the acquisition efficiency of hemostasis skills (30, 31).

VR models for complex HBP surgeries remain limited due to two critical challenges: anatomical complexity and force feedback deficiencies. The intricate vascular and ductal networks of the liver and pancreas demand submillimeter precision in VR modeling, which remains technically challenging. Current haptic devices fail to replicate the viscoelastic properties of HBP tissues, compromising training validity. Technological evolution is addressing these gaps. Next-generation haptic gloves now incorporate variable resistance mechanisms that differentiate between different tissues such as cystic plate dissection (crisp, layered feedback) and liver parenchyma manipulation (mushy, gradual resistance) (32).

Cloud-based VR platforms further enable remote proctoring, allowing experts to virtually observe trainee performance and annotate errors in real-time—a feature particularly valuable for low-resource settings (33). Nevertheless, motion sickness persists in a subset of users during prolonged VR sessions, underscoring the need for improved ergonomic design (34).

Although VR systems can achieve high-fidelity scene simulation in the visual and auditory dimensions, they have obvious shortcomings in the training of delicate surgical operations such as suturing, dissection and aneurysm clipping, due to limitations like the lack of tactile feedback and the required learning and adaptation period for the equipment. To break through the limitations of traditional interaction methods, research on the application of gesture recognition technology in the field of virtual surgery has gradually deepened.

Zhao et al. developed a gesture recognition-based virtual surgery prototype system, which integrates virtual reality technology, gesture recognition algorithms, collision detection models and biomechanical models of maxillofacial soft tissues to realize real-time virtual surgical operations driven by hand gestures (35). Ferreira et al. developed a 3D virtual hysteroscopic surgery simulator based on Leap Motion gesture interaction technology, which captures the movement trajectories of hands and fingers to simulate the basic operation procedures of hysteroscopic surgery. Relevant research findings indicate that the gesture interaction mode can significantly enhance the interactivity and immersion of virtual surgical training, whereas the corresponding simulators for HBP surgeries remain to be developed (36).

## 4 AR systems

AR has emerged as a transformative tool in HBP surgical simulation training, offering distinct advantages over traditional methods. This technology allows for the superimposition of digital information onto the real world, providing surgeons with unique perspectives that can improve surgical precision and outcomes.

One of the key benefits of AR in surgical training is its ability to provide real-time, image-guided assistance during procedures. This capability is particularly valuable in HBP surgeries, where precise navigation and identification of anatomical landmarks are crucial. AR systems can overlay critical information, such as the location of tumors or vascular structures, directly onto the surgical field, thereby improving the surgeon's spatial awareness and decision-making capabilities (37, 38). By integrating holographic anatomical models with real-time clinical data, AR enhances trainees’ spatial understanding of complex liver structures, such as hepatic vasculature and pancreatic ductal systems, enabling precise preoperative planning and intraoperative navigation (39, 40). Studies demonstrate that AR-based simulation improves trainees’ ability to interpret 3D CT/MRI reconstructions, refine resection strategies, and practice anastomotic techniques in realistic virtual environments (41).

Moreover, AR has shown promise in reducing the learning curve for complex surgical procedures. By providing a realistic and immersive training environment, AR allows trainees to practice and refine their skills without the risks associated with live surgery. This is particularly beneficial in HBP surgery, where the complexity of the procedures requires extensive training and experience. Studies have shown that AR can significantly improve the proficiency of novice surgeons, enabling them to achieve better outcomes in a shorter period (42, 43).

AR's immersive nature also facilitates remote collaboration and real-time mentorship. Through shared holographic interfaces, experts can guide trainees across geographical distances, annotating critical structures or correcting errors during simulated procedures (44). This interactive approach bridges the gap between theoretical knowledge and practical skill, particularly in minimally invasive surgeries like laparoscopic liver resection, where haptic feedback limitations in traditional simulators are mitigated by AR's dynamic visual cues (45).

Systematic reviews further validate AR's educational efficacy, reporting improved surgical competency, reduced complication rates, and enhanced trainee confidence compared to conventional training modalities (46). As an evolving technology, AR continues to redefine HBP surgical education by fostering scenario-based learning, improving anatomical precision, and democratizing access to specialized training.

Despite the promising applications of AR in HBP surgical training, there are still challenges to be addressed. The accuracy and reliability of AR systems depend on the quality of the tracking and registration processes, which can be affected by factors such as organ movement and deformation during surgery. Ongoing research and technological advancements are needed to overcome these limitations and further enhance the effectiveness of AR in surgical training (47, 48). The cognitive load imposed by simultaneous holographic and laparoscopic viewing remains a significant hurdle and requires further resolution (49). AR simulation training still has a long way to go in achieving intelligent, precision-based, and human-centered capabilities.

## 5 Discussion

The rapid adoption of minimally invasive HBP surgery has necessitated a paradigm shift in surgical education and training, moving beyond apprenticeship models to technology-enhanced simulation (50). Our analysis reveals distinct yet complementary roles for existing modalities. Animal models retain irreplaceable status for whole-procedure rehearsal, particularly in hemorrhage control scenarios where synthetic models lack physiological responsiveness (51). Yet ethical concerns and regulatory restrictions drive demand for alternatives, spurring development of other existing modalities (Table 1).

**Table 1:** Qualitative comparison of simulation modalities.

Modality	Educational strengths	Key limitations	Innovation frontiers	Typical evaluation metrics	Validation level
Animals	Live tissue response, crisis training	Complex preparation, ethical concerns	Perfused ex-vivo system	Decision-making efficiency, hemorrhage control success rate, team coordination	Construct validity (human-like physiological response); transfer validity (clinical crisis skill translation)
3D printing	Tactile anatomy replication	Static physiology simulation	Bioprinting technologies	Suture accuracy, procedural time, anastomosis quality, expert scoring	Content validity (anatomical fidelity); construct/transfer validity (skill improvement, reduced clinical complications)
VR	Customizable scenarios, performance analytics	Haptic feedback, motion sickness	New-generation haptic tools	Motion/force metrics, error rates, task time, OSATS/GOALS-like scoring	Construct validity (novice-expert differences); predictive/transfer validity (OR skill translation)
AR	Real-time anatomical guidance	Device ergonomics, latency issues, cognitive load	Intelligent, precision-based, and human-centered capabilities	Anatomical identification accuracy, resection precision, trainee competency scores	Construct validity (spatial reasoning improvement); transfer validity (enhanced clinical surgical precision)

OSATS, objective structured assessment of technical skills; GOALS, global operative assessment of laparoscopic skills.

3D-printed models excel in replicating patient-specific anatomical variations. 3D printing has revolutionized surgical education by providing a powerful tool for simulation-based training. Its ability to create accurate, patient-specific models enhances the understanding of complex anatomical structures and improves surgical planning and execution (14–18). As this technology continues to evolve, it is likely to play an increasingly important role in the training of future surgeons, particularly in complex fields such as laparoscopic hepatobiliary and pancreatic surgery. However, their static nature fails to simulate intraoperative dynamics such as respiratory motion or pulsatile blood flow, limiting their utility in crisis management training (22). Recent innovations in bioprinting with live hepatocytes (20) and self-healing vascular networks (5) suggest pathways toward dynamic physiological simulation, though material durability and cost remain barriers (23). Notably, the integration of customized resistive stretching sensors into 3D-fabricated vessel models has been shown to effectively measure tissue deformation in real-time, laying the groundwork for combining static anatomical realism with dynamic performance feedback (52).

The use of VR simulation in laparoscopic hepatobiliary and pancreatic surgery training offers numerous benefits, including risk-free skill development, improved skill transferability, and enhanced patient safety. In addition to skill acquisition, VR simulation also plays a role in reducing the learning curve associated with complex surgical procedures (29). By providing a platform for trainees to practice and perfect their skills, the likelihood of errors during actual surgeries is reduced, thereby enhancing patient outcomes. This advantage is echoed in high-fidelity physical simulators for robotic vascular surgery, which allow trainees to practice vessel isolation, loop placement, and stapler insertion under realistic anatomical constraints without risking patient harm (52). Nevertheless, the current generation of haptic interfaces inadequately replicates the viscoelastic properties of surgical tissues. Next-generation solutions, such as magnetic levitation haptic gloves with tissue-specific resistance profiles (32), show promise but require validation against intraoperative force measurements. Preliminary validation of sensorized vascular simulators has demonstrated construct validity, with expert surgeons applying significantly lower vessel strain than novices—a metric directly linked to reduced bleeding risk in clinical practice (52).

AR's unique value lies in its ability to superimpose virtual anatomy onto real-world environments, enhancing spatial reasoning during complex resections. Clinical studies confirm that AR navigation improves surgical identification accuracy (37–40). However, the cognitive load imposed by simultaneous holographic and laparoscopic viewing remains a significant hurdle (49). Augmented reality represents a significant advancement in HBP surgical simulation training, offering numerous benefits in terms of educational effectiveness and surgical precision. As technology continues to evolve, AR is poised to play an increasingly important role in the training and development of future surgeons, ultimately may improve patient outcomes and enhanced surgical care in selected setting.

To strengthen validation and objective assessment, multiple simulation modalities have demonstrated favorable content, construct, and predictive validity, with increasing emphasis on objective performance metrics including motion tracking, applied force, and procedural error rates. These quantitative measures provide a robust bridge between simulation performance and measurable surgical skill acquisition and transfer (53, 54) (Table 1).

Modern VR/AR platforms enable highly immersive training environments and scalable repetitive practice without patient risk. Complementing these virtual technologies, sensorized physical simulators can provide quantitative feedback on instrument handling and applied forces, supporting standardized and multisite assessment of surgical performance. In this context, the sensorized vascular high-fidelity physical simulator introduced by Gamberini, G.; et al. has demonstrated feasibility and reliability for robot-assisted surgery training in a multisite pilot evaluation, highlighting the value of quantitative, objective metrics in surgical skills assessment (55).

Future hybrid simulation systems could combine AR's virtual anatomical overlays with the tactile realism and objective feedback of sensorized physical simulators, creating a comprehensive training platform for complex HBP and vascular procedures (52).

## 6 Conclusion

With the rapid development of computer technologies [including VR, AR, and artificial intelligence (AI)], innovative ideas have been provided for medical diagnosis, treatment, and medical education that break away from traditional clinical scenarios. Centering on the goals of medical education and talent cultivation, and focusing on complex hepatobiliary and pancreatic anastomosis surgeries, it is an urgent problem to be solved at this stage to help surgeons perceive the teaching context of surgical skills and optimize teaching modes by means of information technology and artificial intelligence, and to realize the transfer and reproduction of laparoscopic surgical experience and skills through personalized assessment and precision teaching.

Simulation-based training is redefining mastery in laparoscopic HBP surgery, offering solutions to the ethical, logistical, and technical constraints of traditional training. While no single modality fully replicates the operating environment, strategic integration of 3D printing's tactile realism, VR's procedural adaptability, AR's spatial contextualization, and animal models’ physiological fidelity can create comprehensive training ecosystems. As simulation technologies evolve, they must remain anchored to surgical education's ultimate goal: ensuring every patient benefits from a surgeon's skills being perfected first in the simulated realm.
